# Waveguide grating couplers with bandwidth beyond 200 nm

**DOI:** 10.1515/nanoph-2024-0683

**Published:** 2025-03-07

**Authors:** Xuetong Zhou, Ying Xue, Hanke Feng, Jianfeng He, Xiankai Sun, Cheng Wang, Kei May Lau, Hon Ki Tsang

**Affiliations:** Department of Electronic Engineering, 105827 The Chinese University of Hong Kong, Shatin, Hong Kong, China; Department of Electronic and Computer Engineering, Hong Kong University of Science and Technology, Clear Water Bay, Kowloon, Hong Kong, China; Department of Electrical Engineering & State Key Laboratory of Terahertz and Millimeter Waves, City University of Hong Kong, Kowloon, China

**Keywords:** integrated optics, photonic integrated circuit, grating couplers, silicon photonics, silicon on insulator technology

## Abstract

We propose and validate a new approach for wideband waveguide grating couplers (GC). The wideband operation is achieved using a slot waveguide grating structure above the conventional channel waveguide. With this slot waveguide grating structure, both the grating strength, mode effective index and dispersion in the grating region can be flexibly tuned to enable high coupling efficiency and wideband operation. 3D FDTD simulations predicted coupling efficiency of −4.08 dB with unprecedented 1 dB bandwidth of 229 nm. The experimental result in coupling with standard single mode fiber in the C band to a lithium niobate waveguide achieved −4.47 dB coupling efficiency with 1 dB bandwidth of 171 nm and 3 dB bandwidth of over 200 nm. The unprecedented wide optical bandwidth is achieved without using bottom metal reflectors or the etching of grating structures on the lithium niobate material.

## 1 Introduction

Photonic integrated circuits (PIC) can integrate various functional passive and active devices into a single chip with high density to realize various powerful and functional systems in a compact chip scale. These compact integrated circuits can process the photonic signal across multiple dimensions or channels at high speed with low power consumption. PIC can be manufactured in high-volume at low-cost, for various applications including communications, imaging, optical sensing, quantum information processing, photonic neural networks, and photonic signal processing. A common requirement in all these applications is the interface with optical fibers: typical requirements include low loss, wideband optical bandwidth coupling, good manufacturing fabrication tolerances, and tolerance to mechanical misalignment of the fibers with the chip.

Edge couplers and grating couplers are the most widely used approaches for fiber-chip interfaces. Grating couplers (GCs) offer several advantages over edge couplers, including compatibility with^1^ wafer-scale testing, and the avoidance of the need for waveguide facet polishing. They also typically have better manufacturing tolerances and are more robust to mechanical misalignment during manufacturing and thus they can reduce the cost of the mechanical assembly in photonic packaging. Compact focusing GCs are also well suited for interfacing with multicore fibers, which is not easy to achieve using edge couplers. The flexible positioning on the surface of the chip makes GCs particularly valuable for large-scale photonic integrated circuits with numerous inputs/outputs and limited edge space.

The primary limitation of grating couplers in comparison to edge couplers is their narrow bandwidth. Although volume-manufacturable GCs with sub-decibel coupling loss have been successfully demonstrated using standard fabrication processes in commercial foundries [1] using our recently demonstrated optimized shift pattern overlay method [2], [3], their spectral range remains relatively small, typically with a 1 dB optical bandwidth of only about 30 nm. This narrow operational wavelength range significantly impedes the application of GCs in various photonic technologies, including coarse wavelength division multiplexing (CWDM), wideband frequency comb generation, spectroscopic sensing, imaging, and beam steering applications. While edge couplers can be used for wideband coupling in the aforementioned applications, they require precise alignment and are highly sensitive to environmental variations. For stable and high-precision characterization, a wideband grating coupler is preferred, as it offers relaxed alignment tolerance and is less affected by environmental changes. This is crucial for accurate measurements. Additionally, grating couplers allow testing of the design at the wafer scale without the need for chip dicing, reducing costs and enabling faster verification without the complexities of packaging.

Achieving wideband operation in GCs would not only benefit the applications that operate with a wider wavelength range but also enhance coupling stability across a broader temperature range and improve tolerance to fabrication variations, thereby addressing key challenges in their implementation and expanding their potential applications in integrated photonics.

Various methods have been explored to develop wideband waveguide grating couplers (WGCs), including subwavelength engineering [4], [5], [6], [7], low-index materials platform [8], [9], high numerical aperture (NA) fiber coupling [10], and inverse design methods [11], [12], [13]. However, most of these approaches require small features that are typically fabricated using electron-beam lithography (EBL) [11], [12], [14]. Additionally, their 1 dB bandwidth typically remains below 100 nm, and realizing wide bandwidth while maintaining practical manufacturability remains an ongoing challenge. To solve this problem, we previously developed a mirror-symmetric wideband GC with 1 dB bandwidth over 100 nm using standard commercial foundry photolithography fabrication processes, marking a significant advance in this field [15], [16].

In this paper, we further improve the optical bandwidth of grating couplers, propose and experimentally validate a new and novel approach for wideband waveguide grating coupler design which can attain wideband coupling with unprecedented bandwidth of over 200 nm.

We use a vertical slot structure comprising a thin film of lower index material, implemented here with low-temperature oxide (LTO) deposition, sandwiched between the higher refractive index underlying waveguide layer and the upper overlay layer. The low index slot enables flexible engineering of the effective index and dispersion of the grating coupler. The slot waveguide not only lowers the effective index but also adjusts the dispersion through wavelength-dependent mode coupling and mode distribution. By precisely controlling the thickness of the different layers, the GC’s effective index and dispersion can be optimized for wideband coupling. Besides, the LTO layer thickness can be designed to engineer the grating strength to realize high mode matching for high efficiency coupling.

This paper is the first to propose the use of slot waveguide modes and mode coupling in the GC region for the engineering of wideband grating couplers. Wideband coupling is achieved not just through traditional period and duty cycle optimization, but also by controlling the different layer thickness for mode distribution and mode coupling tuning to engineer both the effective index and dispersion in the GC region for wideband coupling.

The 3D FDTD simulation predicted a coupling efficiency of −4.08 dB with a 1 dB bandwidth of 229 nm for a structure that did not require any use of bottom metal reflector on the lithium niobate platform. We fabricated the grating and measured the coupling efficiency to be −4.47 dB and due to the measurement limitation of our laser, we measured a 1 dB bandwidth of 171 nm and 3 dB bandwidth over 200 nm. The designed GC does not need etching of the lithium niobate (LN), and with its minimum feature size over 210 nm, the design is robust and suitable for large-volume manufacturing using photolithography and dry etching of the polysilicon and the oxide layer only. We implemented the grating coupler on the LN platform. The LNOI has excellent potential for many applications stemming from its large linear electro-optic coefficient and suitability for compact, high-speed, low-loss modulators [17].

## 2 Grating optimization and fabrication

**Figure 1:**
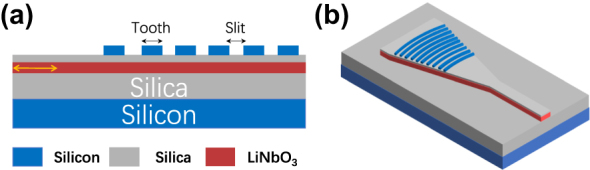
Schematic of the wideband grating coupler. (a) 2D cross-section view. (b) 3D view.

Figure 1 shows the 2D and 3D schematics of the proposed grating coupler on a 300 nm thick Z-cut lithium niobate on insulator (LNOI) with a 2 μm thick buried-oxide layer. A low-temperature oxide (LTO) layer was deposited at 420 °C on the LN layer, followed by a polysilicon layer deposited on the LTO layer via low-pressure chemical vapor deposition (LPCVD) at 620 °C. The grating was formed through a fully etched polysilicon layer. By carefully optimizing the thickness of the LTO and polysilicon layers, along with the grating parameters, both high coupling efficiency and wideband performance can be achieved.

The combination of a high-refractive-index polysilicon layer and an intermediate silicon oxide layer on the LN layer creates a low-refractive-index slot waveguide [18], [19] in the vertical direction. This design can effectively move the mode center from the LN layer upwards into the grating region, and thus increase the grating strength on the low-index contrast LN platform [20], [21]. Careful optimization of the LTO and polysilicon thickness can also improve the directionality of the grating coupler, thus improving the coupling efficiency without the use of bottom mirror or metal reflectors. Additionally, the LTO thickness provides another degree to precise control of the grating strength, enabling good mode matching with better thickness control and high coupling efficiency.

As for wideband GC, due to the diffraction angle of the grating coupler being wavelength dependent and fiber can only receive limited light within its NA, the bandwidth of the grating coupler can be estimated by multiplying the derivative of λ(θ) with a coefficient of 1 dB-bandwidth *η*_1dB_ for single mode fiber (SMF), *η*_1dB_ is about 0.07 for an standard single mode fiber (SSMF) and only depends on the properties of the fiber [5]. Based on the phase matching conditions of the grating coupler:

(1) $$k_{0}n_{\text{eff}}\left(\lambda \right)=k_{0}n_{c}\sin\theta +2\pi q/\Lambda$$

In the Eq. (1) *k*_0_ = 2*π*/*λ* and *n*_eff_ is the effective index in the grating region, *n*_*c*_ is the refractive index of the cladding, *θ* is the diffraction angle of the grating, *q* is the diffraction order (typically equal to 1 for the waveguide grating coupler), Λ is the grating period, and the estimated 1 dB bandwidth can be expressed by

(2) $$\begin{array}{ll} \Delta \lambda_{1d\text{B}} & =\eta_{1d\text{B}}\left|\frac{d\lambda }{d\theta }\right|\\ & =\eta_{1d\text{B}}\left|\frac{-n_{c}\cos\theta }{\left(n_{\mathrm{eff}}\left(\lambda_{0}\right)-n_{c}\sin\theta \right)/\lambda_{0}-dn_{\mathrm{eff}}\left(\lambda \right)/d\lambda }\right|\\ & =\eta_{1d\text{B}}\left|\frac{-n_{c}\cos\theta }{\frac{1}{\Lambda }-\frac{dn_{\text{eff}}\left(\lambda \right)}{d\lambda }}\right| \end{array}$$

And the above equation can also be simplified as below [12],

(3) $$\Delta \lambda_{1d\text{B}}=\eta_{1d\text{B}}\left|-\lambda n_{c}\cos\theta /\left(n_{g}-n_{c}\sin\theta \right)\right|$$

Where $n_{g}=n_{\mathrm{eff}}-\lambda dn_{\text{eff}}\left(\lambda \right)/d\lambda$ , from Eqs. (2) and (3), since $dn_{\text{eff}}\left(\lambda \right)/d\lambda$ is negative as light is weakly confined at longer wavelength. Increasing the grating coupler’s bandwidth can be achieved by reducing the waveguide’s group index, either by lowering the effective index *n*_*eff*_ or reducing the dispersion $-dn_{e\mathrm{ff}\left(\lambda \right)}/d\lambda$ .

Previous approaches primarily relied on subwavelength engineering [5] to reduce the effective index or used low-index material platforms [8] for wideband coupling. However, these methods generally resulted in low coupling efficiency and 1 dB bandwidth of less than 100 nm, while requiring extremely small feature sizes that typically require the use of electron beam lithography and not suitable for volume manufacturing.

Our proposed structure here can help engineer the effective index and dispersion simultaneously and avoid the use of tiny feature sizes, thus making the structure compatible with large-volume manufacturing using the deep-UV photolithography available in many commercial foundries.

As for the dispersion, the light distribution at different wavelengths can be controlled with different layer thickness combinations for dispersion tuning, also the wavelength-dependent mode coupling strength provides another degree to modulate the effective index and dispersion in the GC region for wideband coupling with precise layer thickness control.

In summary, the slot waveguide not only can maintain a high coupling efficiency with a large period, increase the minimum feature size, and easy to be fabricate, but also lower the effective index for wideband coupling, and the wavelength-dependent mode distribution and interaction between modes in the GC region can be flexibly tuned with different layer thickness control [19] for effective index and dispersion engineering in GC region to realize wideband coupling.

Previous designs primarily adjust the period and duty cycle to control the effective index in the grating region, often relying on subwavelength structures to lower the index. However, these approaches require highly precise fabrication, which is difficult with standard photolithography [5]. In our design, we not only adjust the period and duty cycle but also introduce a slot waveguide and vary material layer thickness control to fine-tune the effective index and dispersion for wideband coupling. The introduced mode coupling by the slot waveguide structure in the vertical direction further helps control the effective index and dispersion of the GC, and the coupling strength can be precisely tuned by adjusting the LTO and polysilicon layer thickness for wideband coupling.

The proposed grating coupler design is grounded in fundamental physics, rather than simply adding an extra degree of freedom. The slot structure reduces the effective index (beneficial for wideband operation), improves grating directionality (beneficial for high efficiency coupling without using a bottom mirror) and enable tuning the grating strength for apodization with larger feature sizes (improving fabrication tolerance and manufactuability). The structure can improve the mode matching between the fiber mode and the diffracted mode through additional precise control of the layer thickness instead of the period and duty cycle only method. The slot waveguide pulls part of the light into the low-index LTO layer, lowering the effective index, which is ideal for wideband grating coupler design. By adjusting the thickness of the LTO and polysilicon layers, the effective index and dispersion can be fine-tuned in the GC region, ensuring wideband coupling. Such design have the flexibility to modify the mode distribution and interaction in the grating region, enable modify the effective index and the dispersion [22], [23] simultaneously. In summary, based on this design, both the effective index and dispersion of the grating can be modified simultaneously to realize wideband coupling. If there is no such working principle behind, relying solely on optimization algorithms to achieve such high performance is not possible [24], [25].

**Figure 2:**
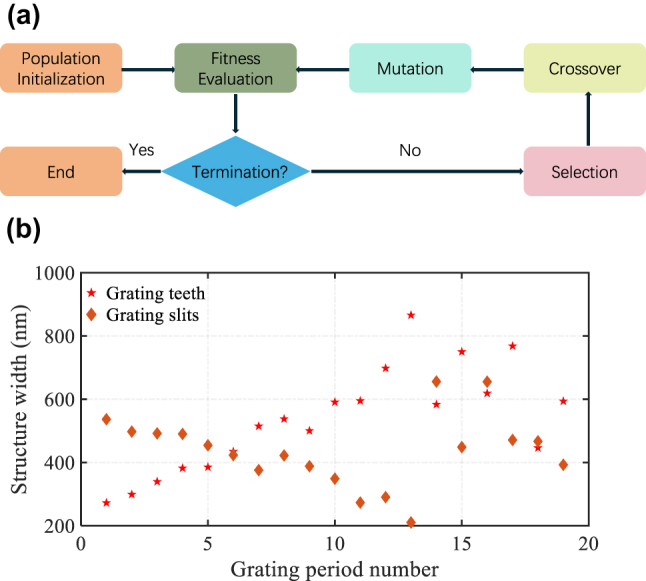
Optimization process and parameters for the GC. (a) Genetic optimization flow chart. (b) Final optimized structural parameters for the wideband GC.

Based on the design principles mentioned, we used a genetic algorithm [26] for numerical optimization of the GC, adjusting the period and duty cycle of the polysilicon overlay. To speed up the process, we used 2D finite-difference time-domain (FDTD) simulations. The genetic optimization algorithm follows these steps, as illustrated in Figure 2(a): First, the algorithm initializes the population by assigning random structural parameters. Next, it evaluates each individual’s fitness through FDTD simulation. The fitness F is defined as: F=Max (CE)*BW(1 dB), where CE represents coupling efficiency and BW represents bandwidth. The algorithm sets a maximum iteration limit and terminates if no improvements occur within 30 generations. It then employs a roulette-wheel selection method to choose individuals based on their fitness scores. The next generation is produced through two genetic operations: crossover and mutation. During crossover, pairs of selected individuals have an 80 % probability of intermixing at a random point, producing two offspring. These offspring then undergo mutation, with a 10 % probability that their structural parameters will be randomly modified. The process forms an optimization loop by returning to the fitness evaluation step, as shown in Figure 2(a). A detailed description of the optimization process can also be found in our previous work [27]. In the simulations, we assumed ordinary (no∼2.21) and extraordinary (ne∼2.14) refractive indices for LiNbO_3_ at 1,550 nm, with index dispersion taken into consideration.

The final optimized parameters are shown in Figure 2(b), with a minimum feature size greater than 210 nm, making fabrication feasible using standard photolithography. The final optimized LTO and polysilicon thicknesses were 200 nm and 270 nm, respectively. The polysilicon layer was fully etched, simplifying the fabrication process, reducing costs, and making the structure less sensitive to etch depth variations. Achieving high coupling efficiency with a fully etched GC is challenging. Typically, a bottom reflector is required to enhance coupling efficiency for fully etched GC, for example, GC can yield −0.58 dB efficiency but needs metal reflector and tiny features difficult to fabricate even with EBL, without the metal reflector, coupling efficiency drops to below −5.5 dB [28].

High-performance grating couplers on LNOI without substrate mirrors is challenging due to the low directionality and low index contrast, especially with the thin (300 nm thickness) LN layer used in this paper. The dry etching of LN is difficult to control, with non-vertical sidewalls being typical, which adversely affects yield and coupling efficiency. In this paper, we implemented the grating coupler using a slot waveguide design formed by depositing low temperature oxide (LTO) on the LN and then depositing a polysilicon layer which is used for defining the GC. The grating structure is only etched in the uppermost polysilicon layer, avoiding the need for high density and deep groove etching in the LN layer and the associated sidewall angle issues. By using transverse magnetic (TM) mode, which has a stronger interaction with the slot waveguide and a lower effective index, we were able to use a larger grating period, improving fabrication tolerance. Combined with reliable dry etching processes for oxide and polysilicon, this approach ensures high yield and makes large-scale production of high-performance GCs feasible [20] on the LNOI platform.

**Figure 3:**
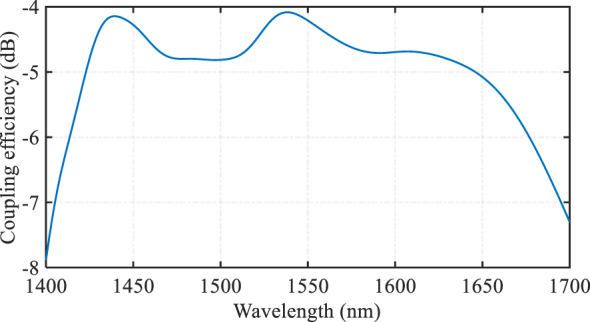
3D FDTD simulated coupling efficiency spectra of the final optimized wideband GC.

The proposed grating coupler is designed for operation with standard single-mode fiber with a mode field diameter of 10.4 μm at 1,550 nm, and for the coupling to the quasi-TM mode of the LN waveguide or vice versa. The 3D FDTD simulation of the structure predicted the coupling efficiency to be −4.08 dB at 1,537 nm, and the 1 dB optical bandwidth to be 229 nm, covering the range from 1,421 nm to 1,650 nm, as shown in Figure 3. The GC is designed for perfectly vertical coupling to simplify the packaging and integration with multicore fibers, lasers, and photodetectors, etc. High directionality can be maintained across a broad bandwidth [29], and the current limitation of low coupling efficiency is primarily due to mode matching [29] with standard single-mode fiber, which has a mode field diameter of 10.4 μm at 1,550 nm, for bandwidths exceeding 200 nm. By narrowing the bandwidth, coupling efficiency could be improved, or alternatively, using a high NA fiber with a smaller mode field diameter. Maintaining high coupling efficiency over a wide bandwidth of 200 nm is a challenging task, particularly for a large mode field diameter of 10.4 μm at 1,550 nm.

**Figure 4:**
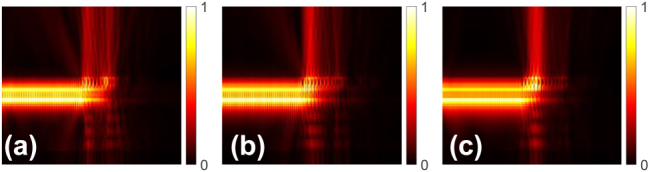
|E| profiles of the wideband GC working at (a) 1,450 nm, (b) 1,550 nm, and (c) 1,650 nm.

Figure 4 shows the cross-sectional view of the |E| profiles for the proposed GC. After the light enters the grating region, the mode moves from the LN layer into the upper region, increasing its interaction with the grating structure. Also, we can see that the GC works well in the wavelength range of 1,450–1,650 nm. The polysilicon layer only changes the mode distribution in the grating region while in the fully etched LN waveguide part, most of the light is confined in the LN layer, making it suitable to utilize the excellent properties of the LN. The proposed GC design not only can be used for Z-cut LN wafers, but also can be used for X-cut LN wafers and other platforms like silicon nitride and silicon platform.

**Figure 5:**
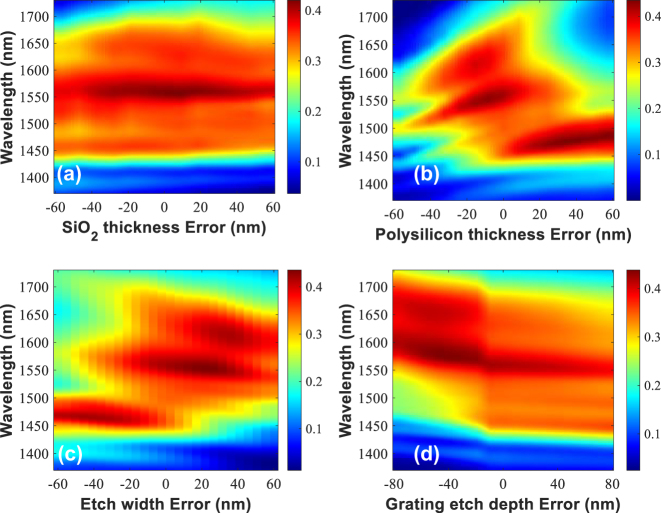
Fabrication tolerance analysis for the GC. (a)–(d) Coupling efficiency spectra versus the fabrication variations for the wideband GC.

We also analyzed the fabrication tolerance of the high-performance GC. The combination of the TM mode and slot waveguide results in a lower effective index and larger grating period, increasing fabrication tolerance. We considered possible variations, including the thickness of the LTO and polysilicon layers, etched depths and widths in the polysilicon layers. The simulated transmission spectra in Figures 5(a)–(f) demonstrate the grating coupler’s robustness to various fabrication variations. The GC maintains high performance despite LTO thickness variations of ±60 nm, polysilicon thickness variations from −20 nm to +40 nm, grating groove etch width variations from −40 nm to +60 nm, polysilicon layer etch depth variations of ±80 nm. All these show that the proposed GC not only shows high efficiency and wideband coupling but also shows fabrication robust properties and potential high performance under volume production.

**Figure 6:**
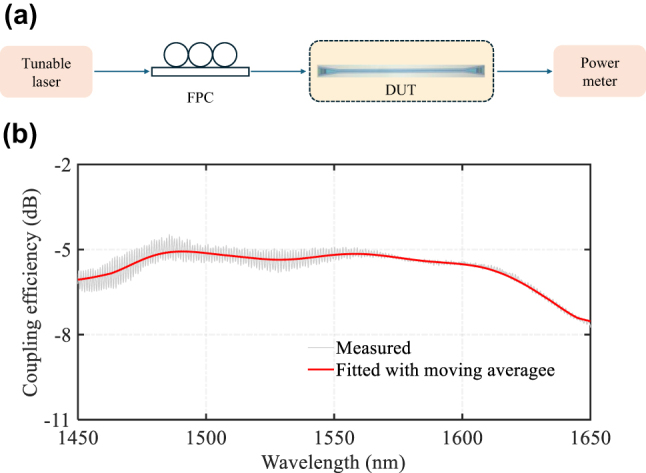
Experimental setup and measurement results for the GC. (a) Diagram of experimental testing. FPC, fiber polarization controller; DUT, device under test. (b) Experimentally measured results for the wideband grating coupler.

We experimentally measured the coupling efficiency of the fabricated grating coupler. Our test devices consisted of two focusing GCs connected by a straight 2 μm wide LN waveguide. Figure 6 shows the experiment setup and the measured transmission spectrum of a single GC. During the experiment, we first measured the transmission spectrum of the two connected grating couplers. After testing the grating couplers, we then measured the insertion loss of the whole link. To maintain consistency, we used the same fiber length that was used in the initial grating coupler tests to connect the two test points, while keeping all other system components unchanged after the measurement of the grating coupler. We then normalized the transmission loss of the link to determine the actual performance of the grating coupler. The final experiment results indicate a peak coupling efficiency of −4.47 dB per grating at 1,485.7 nm, with a 1 dB bandwidth of 171 nm (from 1,450 nm to 1,621 nm). The maximum back reflection of our grating coupler is −12.5 dB at 1,470 nm and the minimum back reflection is −21.6 dB at 1,600 nm, better than the previous inverse designed broadband grating coupler on LNOI with off-vertical coupling [24].

In our experimental results analysis, we assumed negligible waveguide propagation losses and identical performance of input and output GCs. However, fabrication variations might cause different peak coupling efficiency wavelengths at the input and output GCs, potentially leading to better individual GC performance than our averaged measurements suggest. Further optimization of the LPCVD deposition process could improve the thickness and refractive index control of the different layers, potentially enhancing the performance further.

One of the limitations in our setup to measure the optical bandwidth of the waveguide grating is the control of the optical polarization output from the fiber pigtail of the tunable laser. Our measurement first involved optimizing the polarization for the best coupling efficiency at 1,550 nm. But in scanning the laser wavelength from 1,450 to 1650 nm the optical polarization at the output of the fiber may not be fixed for optimum excitation of the TM mode throughout the wide wavelength band, and any deviation of the polarization will adversely degrade the measured coupling efficiency. We therefore think the experimental results presented are a lower bound estimate of the real 1 dB bandwidth and that if we had polarization maintaining fiber for coupling to the GC, we may have a better experiment result than the 171 nm 1 dB bandwidth. Even with this low bound estimate, we still achieved the widest optical bandwidth yet reported using waveguide grating couplers.

During our measurements, we observed that polarization remains stable and well over a wavelength range of more than 100 nm. However, near or beyond 200 nm, coupling efficiency will decrease toward the sidebands (e.g., 1,450 nm and 1,650 nm). Taking this decrease into consideration suggests that the actual bandwidth is broader than the present result. The experimental 1 dB bandwidth of 171 nm is therefore a conservative estimate. Our 3D FDTD simulations predict a 1 dB bandwidth of 229 nm, and considering polarization-related degradation in the sideband, achieving a 1 dB bandwidth exceeding 200 nm, closer to the simulation result, maybe possible.

In comparison with the most recent inverse-designed broadband waveguide grating coupler on lithium niobate, which used a relatively thick 700 nm LN layer to increase coupling efficiency, to −3.8 dB peak coupling efficiency with 1 dB bandwidth of 71.7 nm [24]. Our design employs a thinner 300 nm LN layer, leading to slightly reduced directionality and a coupling efficiency of −4.47 dB with an unprecedented 1 dB bandwidth of 229 nm in simulations and 171 nm in experimental measurements. Besides, the design in [24] required high-density grooves etching with a deep etch depth of 450 nm, leading to sidewall angle issues, our approach also avoids the need for high-density or deep etching of grating layer on LN, thereby mitigating the sidewall angle issues associated with LN etching. We also make a comparison table of the recent grating couplers on LNOI platform, including high efficiency GCs [30], [31], [32], [33], [34], [35] and wideband GCs on LNOI [24], [25], see Table 1.

**Table 1:** Comparison of recent grating couplers on LNOI.

References	Bottom	Overly	Grooves	Exp.	Exp.	Exp.	λ	Angle	MFS	Polarization	Min	Max
			etching	peak	1-dB	3-dB						
	reflector	pattern	on LN	CE (dB)	BW (nm)	BW (nm)	Band	(°)			BR (dB)	BR (dB)
20	NO	Yes	NO	−2.2	…	81	C	5.5	387	TM	…	…
30	YES	NO	YES	−0.89	45	…	C	0	300	TE	−16.5	−13.7
31	YES	Yes	YES	−1.42	…	…	C	1.6	178	TE	…	…
31	YES	Yes	YES	−2.1	…	…	C	3.4	205	TM	…	…
24	NO	No	YES	−3.8	71.7	>120	C	6	175	TE	−18	−10
32	NO	NO	YES	−1.99	…	38	O	2.3	155	TE	…	…
33	NO	NO	YES	−2.81	…	56	C	−22	187	TE	…	…
34	NO	YES	NO	−2.84	…	…	C	10	228	TE	−26	…
35	NO	NO	YES	−2.97	46	…	C	10	155	TE	…	…
25	NO	NO	YES	−3.9	…	90	C	2.5	141	TE	…	…
This work	NO	YES	NO	−4.47	171	>200	C	0	210	TM	−21.6	−12.5

Grooves represent grating grooves; Coupling efficiency (CE); Bandwidth (BW); Minimum feature size (MFS).

Since this work demonstrates an ultrawideband grating coupler with a bandwidth exceeding 200 nm, and considering the trade-off between coupling efficiency and bandwidth, the coupling efficiency of the current grating coupler is not in high level. However, for certain wideband applications, such as CWDM with four channels, a bandwidth of around 80 nm is typically sufficient, meaning the grating coupler does not require a bandwidth over 200 nm, with this relaxed bandwidth requirement, the coupling efficiency of the grating coupler can be improved.

Additionally, to further improve coupling efficiency without compromising performance, complicated subwavelength structures [36] or multi-level etching [37] can be employed. Alternatively, one could use a high NA fiber with a smaller mode field diameter or extend the design to longer wavelength bands, such as 2 µm, where achieving a wideband grating coupler is easier. The proposed grating coupler is well-suited for applications that require wideband coupling but have lower requirements for coupling losses.

The observed discrepancies between the simulated result in Figure 3 and experimental results in Figure 6 can be attributed to various factors, including variations in the refractive indices and thicknesses of the layers during fabrication, potential inaccuracies in assumed material properties used in simulations with actual material properties of LTO and polysilicon in experiment (deposited condition dependent), and process variations in etch depth and width for the grating layer. Despite these challenges, our device achieved the widest bandwidth reported to date. We believe that further optimization of the LTO and polysilicon deposition processes could lead to even better performance, potentially aligning experimental results more closely with simulations and enhancing device performance.

**Figure 7:**
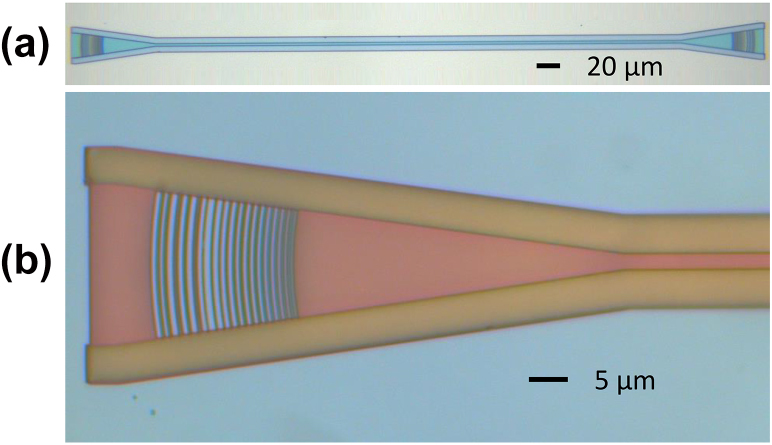
Microscope image of the wideband grating coupler. (a) A pair of GCs. (b) Zoom-in of the single grating.

Figure 7 presents a photograph of the fabricated GC, which employs a focusing grating design [38], [39] to eliminate the need for several hundred micrometers long adiabatic tapers between the narrow channel waveguide and the wide grating region for mode field diameter matching with single-mode fiber. The focusing GC has a compact footprint and is suitable for high-density photonic integrated circuits integration. This proposed design methodology can also be applied to other LN thickness by simply adjusting the thickness of the LTO and polysilicon layers [19]. We demonstrated TM mode here for proof-of-concept demonstration and based on the working principle, this design method can also be used for transverse electric (TE) mode [22], [23].

In this design, the grating is etched only in the polysilicon layer, which avoids the need to etch high density grooves in the LN (lithium niobate) layer and eliminates issues with sidewall angles caused by multiple etchings in LN. The polysilicon grating has a minimum feature size of 210 nm, making it compatible with standard photolithography. This method enables high-efficiency, wideband grating couplers on the LN platform, and better performance might be achieved by etching the LN grating region and using subwavelength structures, the proposed structure can be used to develop high performance nonlinear or modulation devices on LN platforms.

## 3 Conclusions

We proposed and demonstrated a new wideband grating coupler design for standard single-mode fiber in the C-band. By adding a slot waveguide above the conventional grating, the effective index and dispersion can be flexibly tuned for wideband operation. The grating strength is also adjustable through the slot layer thickness, enabling high coupling efficiency and wideband performance. The proposed design offers good fabrication tolerance, with a minimum feature size above 210 nm, making it easy to fabricate in volume using standard photolithography. The design was experimentally validated, and measurements of the fabricated grating showed a peak coupling efficiency of −4.47 dB coupling efficiency with 1 dB bandwidth of 171 nm with this novel method. These results represent the unprecedented wideband operation WGC achieved. This approach will promote the use of GC for wideband operation and open the door for WGC with 1 dB bandwidth toward 200 nm or even 300 nm. The perfectly vertical coupled and compact focusing GC will enable the GC can be integrated with multicore fiber for use with space division multiplexing and wavelength division multiplexing together to increase the transmission capacity in both space and wavelength degree.

We used a standard single-mode fiber for its wide application, through switching to a high-NA fiber could further improve bandwidth. The design was demonstrated on an LN platform due to its wide transparency window and excellent electro-optic and nonlinear properties. However, it can also be applied to platforms like silicon nitride or silicon and adapted for other wavelengths, such as 2 µm, for even greater bandwidth. This method can also be extended to multimode grating couplers for WDM and mode division multiplexing (MDM) combined applications. The demonstration on the LN platform avoided etching the grating layer in the lithium niobate, eliminating sidewall angle issues and ensuring consistent high performance for large-scale production. This design will pave the way for the wideband application of the emerging LiNbO_3_ platform and leverage the hybrid advantages of both silicon and LiNbO_3_. This design method can also be applied to other platforms, such as silicon nitride [22], [23], silicon [29], etc.

Such a wideband GC can help the PIC to unlock a much wider wavelength spectrum for various applications in various PIC platforms. The GC features a wide, flat transmission spectrum and is suitable for large-scale manufacturing. It can be applied to wideband CWDM transceivers, spectroscopic sensing, integrated optical frequency comb sources, beam steering, wideband optical spectrometers, and other wideband applications.

## References

[1] Zhou X., Tsang H. K. (2022). Photolithography fabricated sub-decibel high-efficiency silicon waveguide grating coupler. *IEEE Photonics Technol. Lett.*.

[2] Zhou X., Tsang H. K. (2022). Optimized shift-pattern overlay for high coupling efficiency waveguide grating couplers. *Opt. Lett.*.

[3] Zhou X. T., Tsang H. K. (2022). High efficiency multimode waveguide grating coupler for few-mode fibers. *IEEE Photonics J*..

[4] Xiao Z., Luan F., Liow T. Y., Zhang J., Shum P. (2012). Design for broadband high-efficiency grating couplers. *Opt. Lett.*.

[5] Chen X., Xu K., Cheng Z., Fung C. K., Tsang H. K. (2012). Wideband subwavelength gratings for coupling between silicon-on-insulator waveguides and optical fibers. *Opt. Lett.*.

[6] Cheng Z., Chen X., Wong C. Y., Xu K., Tsang H. K. (2012). Broadband focusing grating couplers for suspended-membrane waveguides. *Opt. Lett.*.

[7] Xu X., Subbaraman H., Covey J., Kwong D., Hosseini A., Chen R. T. (2013). Colorless grating couplers realized by interleaving dispersion engineered subwavelength structures. *Opt. Lett.*.

[8] Doerr C. R., Chen L., Chen Y. K., Buhl L. L. (2010). Wide bandwidth silicon nitride grating coupler. *IEEE Photonics Technol. Lett.*.

[9] Sacher W. D. (2014). Wide bandwidth and high coupling efficiency Si3N4-on-SOI dual-level grating coupler. *Opt. Express*.

[10] Xiang C., Tsang H. K., Cheng Z., Chan C.-K. (2014). Increasing the grating coupler bandwidth with a high numerical-aperture fiber. *11th International Conference on Group IV Photonics (GFP)*,.

[11] Sapra N. V. (2019). Inverse design and demonstration of broadband grating couplers. *IEEE J. Sel. Top. Quantum Electron.*.

[12] Wang Y. (2015). Design of broadband subwavelength grating couplers with low back reflection. *Opt. Lett.*.

[13] Mak J. C. C., Wilmart Q., Olivier S., Menezo S., Poon J. K. S. (2018). Silicon nitride-on-silicon bi-layer grating couplers designed by a global optimization method. *Opt. Express*.

[14] Zhong Q. (2014). Focusing-curved subwavelength grating couplers for ultra-broadband silicon photonics optical interfaces. *Opt. Express*.

[15] Zhou X. T., Tsang H. K. (2024). Photolithography fabricated broadband waveguide grating couplers with 1 dB bandwidth over 100 nm. *IEEE Photonics J*..

[16] Zhou X., Wang Y., Zhang Z., Tsang H. K. (2024). Resonance-enhanced wideband grating for efficient perfectly vertical coupling. *IEEE Photonics Technol. Lett.*.

[17] Wang C. (2018). Integrated lithium niobate electro-optic modulators operating at CMOS-compatible voltages. *Nature*.

[18] Xu Q., Almeida V. R., Panepucci R. R., Lipson M. (2004). Experimental demonstration of guiding and confining light in nanometer-size low-refractive-index material. *Opt. Lett.*.

[19] Zhou X., Zhang T., Chen L., Hong W., Li X. (2014). A graphene-based hybrid plasmonic waveguide with ultra-deep subwavelength confinement. *J. Lightwave Technol.*.

[20] Zhou X. (2023). High coupling efficiency waveguide grating couplers on lithium niobate. *Opt. Lett.*.

[21] Zhou X. (2023). High coupling efficiency waveguide grating couplers for bound-states-in-the-continuum waveguide on lithium niobate. *24th European Conference on Integrated Optics*.

[22] Mak J. C. C., Sacher W. D., Ying H., Luo X., Lo P. G., Poon J. K. S. (2018). Multi-layer silicon nitride-on-silicon polarization-independent grating couplers. *Opt. Express*.

[23] Kohli M. (2023). C- and O-band dual-polarization fiber-to-chip grating couplers for silicon nitride photonics. *ACS Photonics*.

[24] Xie Y., Nie M., Huang S.-W. (2024). Inverse-designed broadband low-loss grating coupler on thick lithium-niobate-on-insulator platform. *Appl. Phys. Lett.*.

[25] He X., Sun D., Chen J., Shi Y. (2024). Inverse designed grating coupler with low loss and high bandwidth on LNOI platform. *IEEE Photonics J*..

[26] Mitchell M. (1998). *An Introduction to Genetic Algorithms*.

[27] Zhou X., Tsang H. K. (2021). Multimode waveguide grating couplers for mode division multiplexing in multi-mode fibers. *2021 Asia Communications and Photonics Conference (ACP)*.

[28] Ding Y., Peucheret C., Ou H., Yvind K. (2014). Fully etched apodized grating coupler on the SOI platform with -0.58 dB coupling efficiency. *Opt. Lett.*.

[29] Zhou X. (2024). Fully etched low-back-reflection and high-efficiency silicon waveguide grating couplers with minimum feature size above 260 nm. *J. Lightwave Technol.*.

[30] Chen B. (2022). Low-loss fiber grating coupler on thin film lithium niobate platform. *APL Photonics*.

[31] Kang S. (2020). High-efficiency chirped grating couplers on lithium niobate on insulator. *Opt. Lett.*.

[32] Huang J., Chen N., Chen K., Chu T. (2024). Low-loss grating coupler with a subwavelength structure on a thin-film lithium niobate substrate. *Opt. Lett.*.

[33] Liao R. (2024). High-efficiency thin-film lithium niobate apodized grating coupler utilizing negative diffraction angle. *Opt. Express*.

[34] Xiong N., Wang J., Yang H., Ma B., Zou W. (2024). High-efficiency dual-level heterogenous grating coupler on CMOS-compatible silicon-lithium niobate platform. *Appl. Phys. Lett.*.

[35] Fang S. (2024). Design and fabrication of a sub-3dB grating coupler on an X-cut thin-film lithium niobate platform. *Opt. Lett.*.

[36] Xia C., Tsang H. K. (2009). Nanoholes grating couplers for coupling between silicon-on-insulator waveguides and optical fibers. *IEEE Photonics J*..

[37] Alonso-Ramos C., Cheben P., Ortega-Moñux A., Schmid J. H., Xu D. X., Molina-Fernández I. (2014). Fiber-chip grating coupler based on interleaved trenches with directionality exceeding 95 %. *Opt. Lett.*.

[38] Waldhausl R., Schnabel B., Dannberg P., Kley E. B., Brauer A., Karthe W. (1997). Efficient coupling into polymer waveguides by gratings. *Appl. Opt.*.

[39] Cheng Z., Chen X., Wong C. Y., Xu K., Tsang H. K. (2012). Apodized focusing subwavelength grating couplers for suspended membrane waveguides. *Appl. Phys. Lett.*.

